# Frontoparietal, Cerebellum Network Codes for Accurate Intention Prediction in Altered Perceptual Conditions

**DOI:** 10.1093/texcom/tgab031

**Published:** 2021-04-23

**Authors:** L Ceravolo, S Schaerlaeken, S Frühholz, D Glowinski, D Grandjean

**Affiliations:** 1 Neuroscience of Emotion and Affective Dynamics Lab, Department of Psychology and Educational Sciences and Swiss Center for Affective Sciences, University of Geneva, Geneva, Switzerland; 2 Department of Psychology, University of Zurich, Zurich, Switzerland; 3 Department of Psychology, University of Oslo, Oslo, Norway

**Keywords:** communication, functional connectivity, intention, neuroimaging

## Abstract

Integrating and predicting the intentions and actions of others are critical components of social interactions, but the behavioral and neural bases of such mechanisms under altered perceptual conditions are poorly understood. In the present study, we recruited expert violinists and age-matched controls with no musical training and asked them to evaluate simplified dynamic stimuli of violinists playing in a *piano* or *forte* communicative intent while undergoing functional magnetic resonance imaging. We show that expertise is needed to successfully understand and evaluate communicative intentions in spatially and temporally altered visual representations of musical performance. Frontoparietal regions—such as the dorsolateral prefrontal cortex and the inferior parietal lobule and sulcus—and various subregions of the cerebellum—such as cerebellar lobules I-IV, V, VI, VIIb, VIIIa, X—a re recruited in the process. Functional connectivity between these brain areas reveals widespread organization, particularly in the dorsolateral prefrontal cortex, inferior frontal gyrus, inferior parietal sulcus, and in the cerebellum. This network may be essential to successfully assess communicative intent in ambiguous or complex visual scenes.

The human ability to coordinate with others is a key evolutionary skill that enables us to accomplish tasks that would otherwise be impossible to manage individually. In addition to vocal and semantic communication (Kotz and Schwartze 2010), such a mechanism relies on finely tuned nonverbal expressive behaviors that must be able to communicate one’s intention reliably and efficiently (Jahng et al. 2017). Intention therefore involves the entire body as a means of communication, with a focus on upper body actions and movement dynamics (Andersen and Cui 2009). It requires that both parties share similar representations at different levels (e.g., a common goal or intermediate steps to reach a final goal), predict common outcomes, and integrate the predicted consequences of their own actions as well as those of others (Sebanz and Knoblich 2009). Thus, coordination also requires paying attention to one’s own intentions and being able to predict and anticipate movement generation (Lau et al. 2004). However, the neural processes underlying such a flow of communication are not yet fully understood. Currently, the literature suggests the use of an internal forward model that optimizes one’s motor control by comparing the actual and predicted sensory consequence of movements (Wolpert et al. 2003b). More broadly, such models could also be used to predict the actions of others in social interactions based on one’s own action representations (Wolpert et al. 2003b). Such abilities would be supported by multiple interacting brain networks that are active in both generating actions and observing the actions of others (Rizzolatti et al. 1996). The frontoparietal network, including the inferior parietal lobe (IPL), has been associated with observation of individual movements (in both monkeys and humans), joint visual attention, and motor intention recognition (Rizzolatti and Sinigaglia 2010). Attention to one’s intentions and actions also recruits the frontoparietal network, particularly the prefrontal cortex, which has strong functional coupling with the premotor cortex (Lau et al. 2004). Another brain region, the posterior parietal cortex, has also been repeatedly observed in situations involving motor intention and imagination (Jeannerod 1994), while regions in the lateral parietal cortex, such as the intraparietal sulcus (IPS) and IPL, have been more directly and precisely linked to attention and intention itself (Lau et al. 2004; Desmurget and Sirigu 2012; Eskenazi et al. 2015). While such a simulation system is described as the mirror neuron system in monkeys based on single-cell recordings, its existence in humans is still controversial (Keysers 2009; Turella et al. 2009; Campbell and Cunnington 2017). Intention recognition is particularly influenced by contextual information and prior knowledge (Brass et al. 2007). It is subject to information availability and processing (whole-body view vs. covert view), context richness (familiar vs. novel context) and expertise in a given task (Rizzolatti and Sinigaglia 2010). A few human studies have highlighted the influence of expertise on the prediction of movements in groups of experts, such as athletes (Aglioti et al. 2008), by showing their improved ability to anticipate the outcome of a given action in others. Compared to sport athletes, musicians have expertise in both specific sensorimotor skills and social signal analysis, and show structural brain changes, particularly in temporal and premotor cortex, as a strong biological basis for their expertise and training (Münte et al. 2002).

While music provides a unique solution to balance a rigorous experimental approach and ecological testing of cognition and high brain function (D’Ausilio et al. 2015), it also provides an excellent playground to explore a more refined form of coordination, joint action, and intention. Musical ensemble performance requires real-time interpersonal coordination at the level of sensorimotor, cognitive, emotional, and social processes (Keller et al. 2014). It involves nonverbal communication of information about musical structure and expressive intentions through the sounds and body movements of the performers. Musical coordination improves when co-performers share common representations of musical goals enabled by internal models in the central nervous system, as it helps them produce their parts in a way that is compatible with the other musicians and helps them anticipate the timing of each other’s actions by generating online predictions during performance (Keller et al. 2014). In his 2016 review, Keller proposed that joint action and coordination in musical ensemble occur in a three-stage process: 1) integrating information related to one’s own part, the parts of others, and the outcome of the joint action while maintaining a distinction between self et al., 2) representing self, others, and the joint action in predictive internal models, and 3) recruiting the motor system to simulate self- and other-produced actions at multiple hierarchical levels. “Self” models aid in the production of one’s own movement by allowing efficient action planning and execution while running slightly ahead of the movement to anticipate and correct potential errors before they occur (Wolpert and Kawato 1998; Jeannerod 2001). The “other” models simulate the observed actions of fellow players and allow a musician to predict what another will do, as well as how and when they will do it (Wolpert et al. 2003a; Keller 2008, 2012). The “other” models help to understand the intentions of the other (Wilson and Knoblich 2005; Schubotz 2007). Finally, the “joint” internal models integrate the outputs of “self” and “other” internal models and then modify their “own” inverse models to compensate for any discrepancies between these outputs (Keller et al. 2016). These internal models originate in the cerebellum, from where they communicate with other brain regions (Ito 2008). They drive simulations of goal-directed actions by recruiting brain areas normally involved in action execution and observation, but without causing overt movement and in the absence of appropriate sensory input (Pezzulo et al. 2013). Internal models are formed and adapted during repetition of a movement and enable us to move skillfully after repeated practice (Ito 2008). Therefore, “self,” “other,” and “joint” internal models must be trained for complex skills such as music making. In particular, the sensorimotor transformations represented in internal models must be acquired, reinforced, and refined through active experience and observational learning (Wolpert et al. 2003a; Schubotz 2007; Cross et al. 2009). While practicing instrumental technique contributes to the development of internal models that are recruited in the production of desired sounds, observing, and listening to fellow players leads to the calibration of internal models that represent other’s action systems and allows an individual to learn to simulate another’s playing style (Repp and Keller 2010). Musicians, through years of practice, develop a strong functional association between a musical note, its visual representation, and the movement required to produce it (Zatorre et al. 2007; D'Ausilio 2010).

Music has been used extensively in this regard to study brain processes involved in action representation, particularly focusing on visuomotor (Stewart et al. 2003; Buccino et al. 2004) and audiomotor (Bangert et al. 2006; Baumann et al. 2007; Lahav et al. 2007) processes. Similarly, dance has also been used extensively to study movement perception and production (for a review, see Sevdalis and Keller 2011). In both cases, point-light biological motion (Johansson 1973) has already been used to focus on brain processes driven by visual motion cues of actions (Saygin et al. 2004; Saygin 2007) and audiovisual integration (Brooks et al. 2007; Klin et al. 2009). However, previous studies (Aglioti et al. 2008; Abreu et al. 2012) have not specifically tested the utility of expertise on the recognition of expressive intentions under suboptimal conditions—when information is missing or altered (Rahnev and Denison 2018)—although this should be thoroughly investigated to better define and expand our knowledge on optimal vs. suboptimal perceptual decision making. Our focus on degraded sensory information helps to isolate the potential of kinematic information as a key source for decoding others’ intentions in a very reduced time window, a situation typical of the ones tackled by musician experts during their performance. Indeed, such a line of research would contribute critically to understanding the behavioral and neural mechanisms underlying better coordination among multiple individuals. It would also provide a better understanding of the brain mechanisms that are necessary and sufficient to enable a correct assessment of intended communication. Under suboptimal conditions, the cerebellum could play an important role in accurately predicting intention. This assumption is based on several functions of the cerebellum such as action comprehension, planning, timing, and, most importantly, because of its finely tuned connections with the basal ganglia and cerebral cortex (Caligiore et al. 2017). Indeed, the cerebellum is part of the networks for action observation (Sokolov et al. 2010) and voluntary movement (Hülsmann et al. 2003), and it is also an important hub for timing and rhythm processing (Molinari et al. 2007; Chen et al. 2008). Cerebellar activity is also enhanced during sensorimotor coordination in violinists (Krause et al. 2010) and is recruited extensively by abstract social cognition—such as fine-grained communicative intentions in short segments of more vs. less emphatic pieces—with involved subregions overlapping with sensorimotor cerebellar territories (Van Overwalle et al. 2014; Sokolov 2018). While the critical role of the cerebellum in optimal perceptual conditions of action and intention decoding is emphasized in the literature above, the likelihood of its involvement in suboptimal conditions also appears to be veryhigh.

To shed light on the mechanisms of communicative intention, we recruited violinists and matched control participants who rated the visual dynamics of short pieces of violin solos presented with the violinist as a point-light display (PLD) after motion-capture recordings on an independent group of expert violinists. Communicative intent was materialized by categorizing piano vs. forte intentional gestures in these short pieces of music while continuous brain scans were performed using functional magnetic resonance imaging (fMRI). These short PLD videos were manipulated to include both original (unmodified, but with visual information only) and modified PLD segments: These modified segments included 1) a condition with spatially randomized initial positions of the dots (namely, the “spatial shuffling” condition) and 2) a condition in which the pieces were cropped so that only the first moments of the segments were preserved (namely, the “temporal cropping” condition). Spatial shuffling allowed us to remove information related to the musician’s body while preserving the kinetic energy associated with the movements. Kinetic energy associated with speed is an important component of expressive gestures and music perception (Eitan and Granot 2006; Schaerlaeken et al. 2017). Musicians in a musical ensemble may not have the attentional skills to focus on every detail of each other’s performance. One strategy might be to focus their attention on the movement of the others, as it has been shown that the brain regions responsible for movement decoding (MT/V5) can be modulated by attention (Treue and Maunsell 1996, 1999). The temporal excerpt mimics a different real-world situation in which the musician may not have access to the full unfolding of the musical gestures and therefore strategizes to respond to only a portion of them (usually the preparatory gesture). Based on these two situations, we then compared the performance of violinists and controls in terms of accurate recognition of expressive play for both normal and altered versions of the segments during fMRI. In other words, our experimental paradigm and population were chosen to test the general hypothesis that experts—that is, violinists—would perform better compared to control participants when asked to identify the communicative intent of both the original and altered musical pieces. We, therefore, hypothesized that there would be a clear and consistent performance advantage of expert violinists over control participants in judging the communicative intention of piano vs. forte for both original and modified (temporally cropped and spatially shuffled) pieces of music. With respect to neuroimaging data, we hypothesized increased activity in the frontoparietal network and cerebellum as a function of expertise that might be modulated by interindividual differences. We expected increased activations in these brain regions, particularly for violinists when evaluating altered musical pieces (temporally cropped and spatially shuffled) compared to control participants. Finally, we predicted stronger coupling between the prefrontal and premotor cortex and between the cerebellum and the frontoparietal network for violinists compared to control participants, both when evaluating the communicative intent (piano vs. forte) of original and altered (temporally cropped and spatially shuffled) musical pieces.

## Material and Methods

### Participants

Thirty-seven right-handed participants took part in this study (25 women, 19 experts, 17 violinists, M age = 27 ± 7 years). Two participants repeatedly fell asleep during data collection and were therefore excluded from the final sample (*N* = 35). Participants were comprised of a group of expert violinists who had received at least 8 years of training at a high musical institution (Geneva School of Music) and a control group of non-violinists who had no musical training. While the number of males and females differed significantly between the two groups, (χ^2^(1, *N* = 35) = 4.83, *P* = 0.027), with more female than male violinists, age did not differ between the two groups (*t*(30.26, *N* = 35) = −0.38, *P* = 0.7). However, the variance induced by gender did not seem to affect our models (Supplementary Tables 1 and 2). All participants were naïve to the experimental procedure and material and had no known history of psychiatric or neurological disorders. Finally, all participants reported normal hearing and normal or corrected-to-normal vision (contact lenses or MRI-compatible plastic glasses). This study was conducted in accordance with the protocol, the current version of the Declaration of Helsinki, ICH-GCP, or ISO EN 14155 (as applicable), and all legal and regulatory requirements of the State Ethics Committee of the University Hospital of Geneva.

### Experimental Stimuli: Communicative Intent Task

The complete set of stimuli consisted of 64 videos, repeated four times (256 stimuli in total, 128 *piano* and 128 *forte*) and split into four runs of equal duration (~5 min). Across runs, the stimuli were organized in eight conditions as follows: 1) spatially unmodified, temporally unmodified, and piano (*N* = 32); 2) spatially unmodified, temporally unmodified, and forte (*N* = 32); 3) spatially unmodified, temporally cropped, piano (*N* = 32); 4) spatially unmodified, temporally cropped, forte (*N* = 32); 5) spatially shuffled, temporally unmodified, piano (*N* = 32); 6) spatially shuffled, temporally unmodified, and forte (*N* = 32); 7) spatially shuffled, temporally cropped, and piano (*N* = 32); and 8) spatially shuffled, temporally cropped, and forte (*N* = 32). Temporally cropped conditions had a duration of 1.2 s, while temporally unmodified conditions had a duration of 2.7 s. Inter-trial interval had a mean duration of 1.5 s with a range of [1 s; 2 s]. Stimulus order within a run was pseudo-randomized so that the same condition would not occur three times in arow.

**Figure 1:**
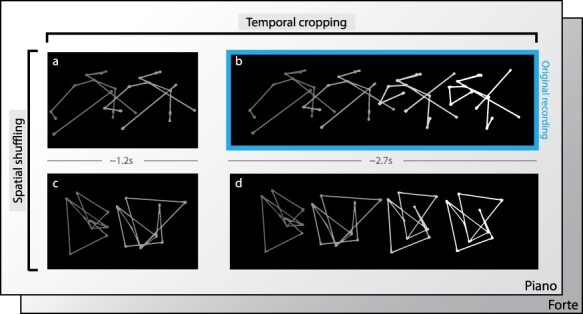
Overview of the stimuli displayed to the participant. The sequence of expressive motion is indicated by a dot-light display based on the collected motion capture of a first violinist of a string quartet. The two temporal segments used in the experiment refer, respectively, to the preparation (temporally cropped condition, ***a***) and the preparation plus the entry part (temporally unmodified condition, ***b***). For each of these temporal conditions, spatial shuffling was also applied as a separate condition (***c* and *d***, respectively). After viewing the selected sequence, participants were asked to indicate the perceived communicative intent of the music (forte or piano) by pressing akey.

These stimuli were produced according to the following procedure. First, using a motion capture system (Qualisys, time sampling), we filmed the first violinist of a professional string quartet during 16 rehearsals of the same piece of music, Death and the Maiden by Schubert, which was chosen because it offers a wide variety of writing and expressive styles. For half of the rehearsals, the first violinist played alone; for the other half, he played with the other members of the string quartet. During the recording sessions, the violinists were instructed to play as expressively as if they were giving a concert performance. The recording sessions took place over two days in a concert hall, as it offers naturalistic conditions perfectly suited to the musicians’ needs and expectations (e.g., the quality of the acoustics). After filming, all clips were processed, and motion capture data were preprocessed to eliminate interfering data through a standard filtering process (despiking and smoothing using MATLAB (The Mathworks, Inc., Natick, MA, USA)) to produce cleaned PLD of the performances. The next step was to select specific moments in each performance where the first violinist indicated his intention to the fellow players with two communicative intentions: piano and forte. To do this, we worked with the violinists to define these key moments and we obtained the corresponding 16 short sequences (8 piano, 8 forte). Two experimental visual manipulations were then applied to each edited point-light sequence. The first manipulation consisted of segmenting the sequence into two parts: the first part referred to the preparation of the entry, just before the generation of the piano or forte gesture (duration = ~1.2 s), and the second part referred to the entire sequence (i.e., movement preparation plus entry (duration = ~2.7 s)). We refer to these sequences as “temporally cropped” vs.” temporally unmodified.” For the temporally cropped sequences, we focused on the first moments of the sequence because we believe it is critical to understand the specific ability of the violinists to coordinate with each other. The second manipulation consisted of destroying the anthropomorphic shape of the stimuli through a single spatial scrambling process that preserved the dynamics of the individual points but shifted their relative relationships to other points. The final shape, which retained the same kinetic energy as the original, had no relation to an anthropomorphic shape. We refer to these sequences as “spatially shuffled” vs. “spatially unmodified.” They were designed to highlight the violinists’ higher processing of dynamic visual information in the absence of visual corporeal anthropomorphic references.

The final excerpts presented to participants were a combination of all three conditions in a pseudorandom order: communicative intent, temporal cropping (Fig. 1*a* and *b*), and spatial shuffling (Fig. 1*c* and *d*). For example, one excerpt might present a piano gesture, and be temporally cropped but spatially unmodified, while another might present a forte gesture, with temporal cropping and spatial shuffling. In all sequences, only visual information was used and the accompanying audio tracks were not presented (see an example for each condition in Fig. 1).

### Experimental Procedure: Communicative Intent Task

Participants, divided into two groups (violinists vs. control participants), were subjected to a 2 (communicative intentions: piano vs. forte) × 2 (temporal cropping: temporally cropped vs. temporally unmodified) × 2 (spatial shuffling: spatially shuffled vs. spatially unmodified) within-subject factorial design, resulting in eight experimental conditions (Fig. 1). Prior to scanning, participants were introduced to and familiarized with the experimental task and confirmed they understood the study and what it required of them. This included performing two trials from each condition (these stimuli were excluded from the fMRI task), while the experimenter monitored performance. During the MRI session, participants watched point-light display movies of the violinist’s movements. After each trial, participants had to report whether the performance related to the communicative intent forte or piano. After the fMRI session, all participants reported that they had no conceptual or technical problem performing the experimental task. The experimental procedure was based on a two-alternative forced-choice task. It aimed to reveal brain mechanisms underlying how groups of violinists (task experts) may differ in enacting fast and accurate decisions in perceptually altered conditions with respect to control participants (non-expert) (Gold and Shadlen 2002; Ratcliff and McKoon 2008; Deneve 2012).

### Behavioral Data Analysis

The statistical software R was used to analyze all behavioral data. We computed a generalized linear mixed model (GLMM) to estimate the variance explained by the piano/forte and violinists/controls fixed factors on the percentage of correct responses. GLMM makes use of random effects modeling to improve the accuracy of the model and to allow the computation of models with non-normal distribution, here a binomial distribution. We tested our predictions for the effect of several fixed effect factors, including participant expertise, communicative intentions, spatial shuffling, and temporal cropping. Random intercept effects encapsulated variability with respect to each participant. We used a step-up strategy in building the model to test the different combinations of fixed effects. Based on the marginality principle, we present the highest order interaction effects (Nelder 1977), namely the interaction between expertise and the other experimental conditions mentioned above. We used chi-square difference tests to examine the contribution of each of our variables and their interactions. We report effect sizes in accordance with the approach of Nakagawa and Schielzeth, implemented in the R package “MuMIn” (Nakagawa and Schielzeth 2013). They created an approach based on two indicators, a marginal and a conditional R2 (R2m and R2c, respectively). R2m is the variance explained by the fixed factors, while R2c is the variance explained by the entire model (both fixed and random effects). These two indicators allow comparability with standard methods, taking into account the variance explained by the random effects. We calculated and reported them for each statistical model.

### Neuroimaging Image Acquisition

Neuroimaging data were acquired using a Siemens Trio 3.0 Tesla MRI scanner at the Brain and Behavioral Laboratory (BBL), University Medical Center, University of Geneva (Geneva, Switzerland). For each participant and for each run of the experimental task, 290 functional T2^*^ -weighted echo planar image volumes (EPIs; slice thickness = 3 mm, gap = 1 mm, 36 slices, TR = 650 ms, TE = 30 ms, flip angle = 90°, matrix = 6464, FOV = 200 mm) were acquired. In addition, a T1-weighted, magnetization-prepared, rapid acquisition gradient echo scan (slice thickness = 1 mm, 176 slices, TR = 2530 ms, TE = 3.31 ms, flip angle = 7°, matrix = 256 256, FOV = 256 mm) was acquired. Thus, 290 volumes with 36 slices were acquired for each participant, resulting in a total of 10 440 slices. The total for all participants was 10,150 volumes and 365,400 slices.

### Neuroimaging Data Analysis

**Figure 2:**
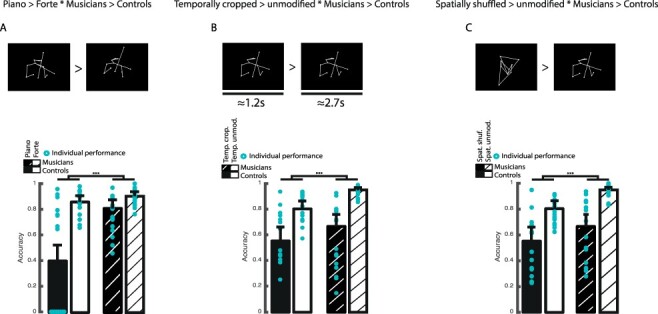
Experimental stimuli and behavioral results for the impact of expertise on intention evaluation. (***a***) Example of piano vs. forte PLD and (***d***) averaged performance and individual points per group for piano vs. forte piece dynamics. (***b***) Example of temporally cropped vs. temporally unmodified PLD and (***e***) averaged performance and individual points per group for piano vs. forte piece dynamics in temporally cropped vs. temporally unmodified sequences. (***c***) Example of spatially shuffled vs. spatially unmodified PLD and (***f***) averaged performance and individual points per group for piano vs. forte piece dynamics in spatially shuffled vs. spatially unmodified sequences. Error bars represent 95% confidence interval. [^*^^*^^*^: *P <* 0.001, ^*^^*^: *P <* 0.01, ^*^: *P <* 0.05;**.**: *P <* 0.1; Spat.: spatial, Temp.: temporal, shuf.: shuffling, crop.: cropping, unmodif.: unmodified].

Functional data were analyzed using Statistical Parametric Mapping version 12 (https://www.fil.ion.ucl.ac.uk/spm) in MATLAB (The Mathworks, Inc., Natick, MA, USA). Preprocessing steps included realignment to the first volume of the time series to correct for head motion, slice timing, normalization to the Montreal Neurological Institute (MNI) template (resampled at 3 × 3 × 3 mm), and finally spatial smoothing with an isotropic Gaussian kernel of 8-mm full width at half-maximum. A high-pass filter of 128 s was used to remove low-frequency components. A general linear model (first-level analysis) was then defined for each participant separately (within-subject statistics). For the experimental task, correctly scored trials were modeled by specific boxcar functions defined by the duration of the video stimuli from stimulus onset to offset and convolved with the canonical hemodynamic response function. Group-level statistics were then performed using a flexible factorial design to account for variance across conditions and participants. Two different group-level models were calculated for the present data: Model 1 included eight conditions (1) spatially unmodified, temporally unmodified, and piano; 2) spatially unmodified, temporally unmodified, and forte; 3) spatially unmodified, temporally cropped, piano; 4) spatially unmodified, temporally cropped, forte; 5) spatially shuffled, temporally unmodified, piano; 6) spatially shuffled, temporally unmodified, and forte; 7) spatially shuffled, temporally cropped, and piano; 8) spatially shuffled, temporally cropped, and forte) and two groups (violinist; control) without covariates [group × conditions], whereas model 2 included task performance as a group-level covariate of interest that interacted with the factor conditions [performance × conditions]. Both models also included a mandatory “participant factor” that allowed for the calculation of between-subject variability. For both group-level models, the specification of independence was set to true for the “participant” and “group” factors, whereas it was set to false for the other factor conditions. Regarding variance estimation, it was set to unequal for all factors including group, because homoscedasticity criteria cannot usually be met for fMRI data (default setting in SPM12). For both models, group-level results in SPM12 were estimated voxel-wise using corrected statistics with *P <* 0.05 false discovery rate (FDR) and an arbitrary cluster threshold of *k* > 10 voxels. For all analyses, regions of significant activation increase were labeled based on probabilistic cytoarchitectonic atlases (Automated Anatomical Labelling Atlas (Tzourio-Mazoyer et al. 2002), Cerebellum Atlas (Diedrichsen et al. 2009; Diedrichsen et al. 2011)), and rendered on semi-inflated brains from the CONN toolbox (http://www.nitrc.org/projects/conn), see Fig. 2.

#### Model 1: Conditions Training Group-Level Statistics

For this first model, regressors were created for each experimental condition and for each participant (*N* = 35), resulting in a first-level planning matrix that contained a total of 16 regressors, including 8 regressors of interest (conditions, see above) and 8 regressors of no interest (incorrect trials, the six motion parameters, and the constant term). Each regressor of interest was used to compute main effect contrast vectors, which were then carried forward into a second-level, group analysis using the flexible factorial design specification that we will describe in detail here. The group-level analysis included the following factors: conditions (see above) and group. The condition factor was used to compare violinists with control participants on their ability to judge the communicative intent of the stimuli, regardless of whether they were spatially shuffled and/or temporally cropped. The following contrasts were therefore computed using the factorial architecture of the data mentioned above: [piano > forte ^*^ violinists > controls], [temporally cropped > temporally unmodified ^*^ violinists > control], and [spatially shuffled > spatially unmodified ^*^ violinists > control] (see Fig. 2).

#### Model 2: Conditions Training Group-Level Statistics with Task Performance as Second-Level Covariate

The second model used exactly the same settings and factorial structure as Model 1, in addition to a group-level covariate that accounted for task performance for each participant (percentage of hits during the experimental task). This covariate was set to interact with the conditions factor. Therefore, Model 2 contained the following factorial structure: condition ^*^ task performance. This model was used to constrain our statistical results and to observe how some brain regions are sensitive to task performance in violinists as opposed to control participants for the following contrasts: [piano > forte ^*^ performance], [temporal cropped > temporal unmodified ^*^ performance], and [spatially shuffled > spatially unmodified ^*^ performance]. The results of this second model have been overlaid in green-to-blue in Figure 2.

### Functional Connectivity Analyses

Functional connectivity analyses were performed using CONN Toolbox v18.a (Whitfield-Gabrieli and Nieto-Castanon 2012). Interfering noise sources were estimated and removed using the automated Toolbox preprocessing algorithm, and the remaining BOLD time series was bandpass filtered using a low frequency window (0.008 < *f* < 0.09 Hz). Correlation maps were then created for each condition of interest by taking the remaining BOLD time course for each condition from the atlas ROIs and calculating bivariate Pearson’s correlation coefficients between the time courses of each voxel of each region of the atlas. These correlations were then converted to normally distributed values using the Fisher transform. Finally, group-level analyses were performed using these Fisher-transformed correlation maps to test for main effects within groups and significant connectivity differences between groups for the contrasts of interest. Type I errors were controlled for by using the seed-level FDR correction with *P <* 0.05 to correct for multiple comparisons.

## Results

### Behavior

Behavioral results showed a generalized and reliable advantage of violinists over control participants in correctly discriminating between the communicative intent piano or forte (Fig. 2). Interaction effects of our factors (group [violinists>controls] and conditions [forte>piano, temporally cropped>temporally unmodified, spatially shuffled>spatially unmodified]) explained a greater proportion of the variance for each statistical model, compared to models with only the main effects (all *P <* 0.001, full statistics in Supplementary Table 2). More specifically, for each computed model, we observed that the performance of all our participants decreased significantly when the information was altered (temporally cropped, spatially shuffled; Fig. 2*b* and *c*) or more subtle (piano, Fig. 2*a*) (all *P <* 0.001, full statistics in Supplementary Tables 2 and 3). At the group level, violinists outperformed control participants in estimating communicative intentions regardless of the condition presented (Fig. 2*a*-*f*, all *P <* 0.001). Finally, we describe a significant interaction effect between group and conditions for each model (all *P <* 0.001). No differences were observed between genders (Supplementary Fig. 1, Supplementary Table 1).

**Figure 3:**
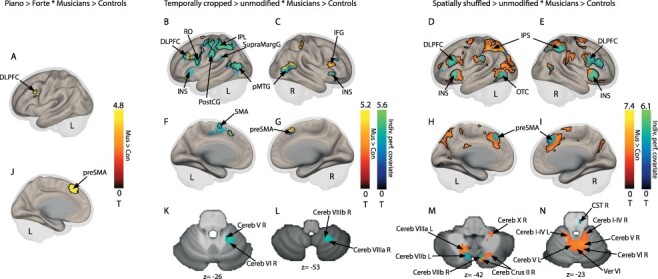
Neural evidence for decoding communicative intentions in violinists and control participants. (***a* and *j***) Increased activity in preSMA and DLPFC for piano vs. forte sequences in violinists vs. control participants. (***b*,*c*,*f*,*g***) Increased activity for temporally cropped vs. temporally unmodified sequences in violinists vs. control participants in red-to-yellow in IPL, and with overall task performance as group covariates in blue-to-green in IPL, DLPFC, pMTG, preMSA., and cerebellar subregions (***k* and *l***). (***d,e,h,i***) Increased activity for spatially shuffled vs. spatially unmodified sequences for violinists vs. control participants in red-to-yellow in IPS, preSMA, DLPFC, and INS, and with overall task performance as group-level covariates in blue-to-green in IPS, preSMA, DLPFC, INS, and OTC and in cerebellum (***m* and *n***). Color bars represent statistical T values of contrast. Black outlines delineate the regions of the group analysis of model 1—not the performance based analysis of model 2. [Cereb: cerebellum lobule; Cereb Crus: cerebellum crus of ansiform lobule; CST: corticospinal tract of the brainstem; DLPFC: dorsolateral prefrontal cortex; IFG: inferior frontal gyrus; INS: insula; IPL: inferior parietal lobule; IPS: inferior parietal sulcus; lingual gyrus; OTC: occipito-temporal cortex; pMTG: medial temporal gyrus; posterior part; preSMA: pre supplementary motor area; PostCG: postcentral gyrus; RO: Rolandic operculum; SupraMargG: supra marginal gyrus; Ver: vermis; Violon.: violinists; Con.: control participants; L: left; R:right]. Voxel-wise *P <* 0.05 FDR corrected.

### Neuroimaging

#### Whole Brain Data

Neuroimaging results focused on regions showing enhanced activations for violinists compared to control participants, when also comparing communicative intention, temporal cropping, and spatial shuffling to unmodified excerpts. Analyses focused exclusively on trials in which participants correctly identified the intention presented in the PLD. When focusing on the excerpts that expressed a piano nuance (piano > forte ^*^ violinists > control), we observed enhanced activations in the pre-supplementary area (preSMA) (MNI coordinates in Supplementary Tables 4 and 5) and left dorsolateral prefrontal cortex (DLPFC) (Fig. 3*a* and *j*, Supplementary Tables 4 and 5, Supplementary Figures 2 and 3). When we focused on the temporally cropped sections (temporally cropped > temporally unmodified ^*^ violinists > control), we observed increased activations in the left IPL, cerebellar lobes V, VI, VIIb, and VIII, bilateral posterior middle temporal gyrus (MTG), and inferior frontal gyrus (IFG) (Fig. 3*b,c,f,g,k,l*; Supplementary Tables 4 and 5, Supplementary Figures 2 and 3). Finally, when we focused on the spatially shuffled excerpts (spatially shuffled > spatially unmodified ^*^ violinists > control), we observed enhanced activations in the bilateral intraparietal sulcus (IPS), the right preSMA, and the bilateral DLPFC, which extended to the IFG pars opercularis and triangularis, the cerebellum (vermis areas IV,V, crus II, lobules I-IV, V, VI, VIIb, VIIIa, VIIIb, X), and the bilateral insula (Fig. 3*d,e,h,i,m,n*; Supplementary Tables 4 and 5, Supplementary Figures 2 and 3). To further highlight these increased activations in the context of each participant’s global performance, we also calculated another second-level analysis with the participants’ individual performance as group-level covariate. Consequently, we were able to account for brain regions with enhanced activations in relation to overall performance (group-level analysis) for our contrasts of interest. These results include interindividual variability with respect to task performance across groups (continuous, one average value per participant). Displayed in green-to-blue activations (Fig. 3*b-e,f-i,k-n*; Supplementary Tables 4 and 5, Supplementary Figure 2), the global individual performance analysis showed a strong overlap with the above brain regions for the temporally cropped condition, particularly in the IPL, SMA, and cerebellum (temporally cropped > temporally unmodified ^*^ violinists > control with individual performance, Fig. 3*b,c,f,g,k,l*; Supplementary Tables 4 and 5, Supplementary Figure 2). This result suggests that this network plays an important role in the accurate assessment of communicative intention of temporally cropped PLD as a function of performance and expertise, and is also sensitive to individual performance differences. This overlap between analyses was smaller for the spatially shuffled excerpts (spatially shuffled > spatially unmodified ^*^ violinists > control with individual performance), especially in the cerebellum. Indeed, the results show that parts of the bilateral insula, bilateral preSMA, bilateral DLPFC, and right IPS overlapped, highlighting the important role of these regions in both performance and expertise. Interestingly, the left IPS showed much less overlap between analyses than the right IPS, suggesting interhemispheric dissociation (Fig. 3*d,e,h,i,m,n*), with left IPS activity massively enhanced in correctly assessed piece dynamics, whereas the right IPS was modulated by interindividual differences in performance. Such an overlap between analyses was also observed bilaterally for cerebellar area VIIb (Fig. 3*m*). Additional brainstem activity was observed in the right corticospinal tract (Fig. 3*n*).

#### Functional Connectivity Data

**Figure 4:**
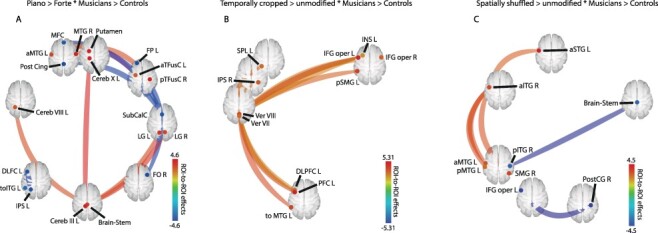
Functional connectivity of decoding communicative intentions in violinists as compared to control participants. (***a***) Increased connectivity for piano vs. forte excerpts in violinists vs. control participants. (***b***) Increased connectivity for temporally cropped vs. unmodified sequences in violinists vs. control participants. (***c***) Increased connectivity for spatially shuffled vs. unmodified sequences for violinists vs. control participants. [aITG: inferior temporal gyrus, anterior part; aMTG: medial temporal gyrus, anterior part; aSTG: superior temporal gyrus, anterior part; aTFus: temporal fusiform, anterior part; Cereb: cerebellum lobule; DLPFC: dorsolateral prefrontal cortex; FO: frontal operculum; FP: frontal pole; IFG oper: inferior frontal gyrus operculum; INS: insula; IPL: inferior parietal lobule; IPS: inferior parietal sulcus; LG: lingual gyrus; MFC: medial frontal cortex; MTG: medial temporal gyrus; PFC: prefrontal cortex; pITG: inferior temporal gyrus, posterior part; pMTG: medial temporal gyrus, posterior part; Post Cing: posterior cingulate; PostCG: posterior central gyrus; pSMG: superior medial gyrus, posterior part; pTFusC: temporal fusiform cortex, posterior part; R: right; SMG: superior medial gyrus; SPL: superior parietal lobule; SubCalC: subcallosal cortex; toITG: inferior temporal gyrus temporo-occipital part; toMTG: medial temporal gyrus, temporo-occipital part; Ver: vermis]. Seed-level *P <* 0.05 FDR corrected.

Atlas-based analyses of seed-to-seed functional connectivity (FC) were performed to highlight the existence of widespread coupled brain activity targeting frontoparietal and cerebellar regions related to both communicative intention processing and expertise. These analyses revealed the involvement of numerous regions observed in our whole-brain contrasts of interest, in addition to subcortical and cerebellar connectivity (Fig. 4). A general effect of expertise across conditions (violinists > control, main effect of all conditions) showed functional coupling between the left IPL and the left postcentral gyrus (Supplementary Fig. 4). As for our contrasts of interest, communicative intention and expertise (piano > forte ^*^ violinist > control) interacted and resulted in both coupled and negatively coupled functional networks (Fig. 4*a*; Supplementary Table 6). Specifically, we observed coupled FC between the bilateral MTG, left putamen, bilateral fusiform cortex, brainstem, and several subregions within the cerebellum, such as cerebellar lobules III, VIII, and X of the left hemisphere. Negatively coupled FC was observed between the medial frontal cortex, posterior cingulate gyrus, frontal pole, left DLPFC, and left IPS (see details in Fig. 4*a*; Supplementary Table 6). For temporally cropped excerpts (temporally cropped > temporally unmodified ^*^ violinists > control), only coupled FC was observed. More specifically, the analyses revealed widespread fronto-parieto-cerebellar FC in the bilateral inferior frontal gyrus pars opercularis (IFGop), left DLPFC, left superior parietal lobule (SPL), right IPS, and vermis areas VII and VIII (see details in Fig. 4*b*; Supplementary Table 6). The final contrast of interest with visually shuffled PLD (spatially shuffled > spatially unmodified ^*^ violinists > control) highlighted coupled FC between the anterior part of the left inferior, middle, and superior temporal gyri (aITG, aMTG, and aSTG), the left posterior MTG, and the right supramarginal gyrus, whereas negatively coupled FC characterized connectivity between the left IFGop, right posterior ITG, posterior cingulate gyrus, and brainstem (see details in Fig. 4*c*; Supplementary Table 6).

## Discussion

The present study aimed to gain a clearer understanding of the interaction between expertise and assessment of communicative intention as a potential proxy for social interactions and coordination. Using point-light representations of violinists as stimuli, we asked experts and non-experts, namely violinists and control participants with no musical training, to rate the expressive intention of the performances. Expressive intent could materialize as piano or forte and could be visually modified, temporally modified, both, or neither. Our results show that violinists consistently performed better than control participants, whether for unmodified or modified stimuli. Premotor and lateral parietal areas together with the dorsolateral prefrontal cortex, as well as numerous cerebellar regions, appear to be the critical players in the violinists’ advantage in successfully assessing communicative intent.

Behavioral results confirmed the role of expertise in perceiving the intentions of others. Violinists were more consistent and accurate in perceiving expressive gestures, indicating a close link between perception and action skills (Wöllner and Cañal-Bruland 2010; Küssner et al. 2014). Importantly, these results held true even when information was absent or altered, highlighting the advantages of action expertise in understanding, integrating, and predicting actions based on the short dynamics of segments and even when anthropomorphic information was altered. Even in the absence of visual contact, musicians have been shown to be capable of interpersonal coordination because they can rely on auditory imagery to promote the operation of internal models that simulate their own and others’ actions during ensemble performance (Keller and Appel 2010). This underscores the importance of combining perceptual information about one’s own and others’ goal-directed movements for action processing and prediction (Spunt and Lieberman 2012).

Our neuroimaging results revealed a widely ramified network of brain regions depending on expertise in decoding or inferring communicative intent, especially under highly altered perceptual conditions. Violinists’ advantage in understanding and estimating communicative intent recruited the DLPFC, which is associated with action observation (Rizzolatti and Sinigaglia 2010) and influenced by training and expertise (Moore et al. 2006), and the preSMA, which is mainly involved in internally and externally selected actions (Mueller et al. 2007). Therefore, preSMA and DLPFC appear to be sufficient for accurately internalizing actions and extracting communicative intentions in experienced participants, respectively. These regions would also explain violinists’ ability to integrate the temporal structure of rhythm via working memory (Chen et al. 2008). Functional connectivity analyses also revealed that expert participants relied on both positively and negatively coupled networks to successfully infer communicative intention. Positively coupled networks include areas involved in intention probability (putamen; Zapparoli et al. 2018) as well as motion prediction and motor imagination (cerebellum; Sokolov et al. 2017) and time encoding (brainstem and cerebellum; Rao et al. 2001; Molinari et al. 2007). More specifically, a large region of the cerebellum, including lobule VIII (and VIIIa), has been shown to covary with instrumental expertise, particularly for temporal complexity (Chen et al. 2008). This finding raises the question of whether general or instrument-specific skills of violinists directly influence cerebellar activity; this important distinction should be investigated in future studies. In a lesion study, lobule III—as well as lobules I, IV, and V—was also involved in action observation (Sokolov et al. 2010), whereas lobule IX and, to a lesser extent, lobule X were repeatedly associated with verbal working memory (Van Overwalle et al. 2014). Thus, as a functionally connected cerebellar network and through connections with the brainstem and basal ganglia, these lobules confer strong weight on the cerebellum as a crucial player in action observation, processing, and prediction. On the other hand, negatively coupled activity recruited brain regions known for mental states attributed to moving shapes and for memory for intentions (medial prefrontal cortex or frontal pole; Blakemore and Decety 2001), for action observation (ITG and DLPFC; Blakemore and Decety 2001; Rizzolatti and Sinigaglia 2010), and for attention to and understanding of intentions (IPS; Blakemore and Decety 2001).

The behavioral advantage in the evaluation of communicative intention under altered perceptual conditions by experienced participants relied essentially on several of the brain areas mentioned above with some additions. In temporally cropped sequences, expertise additionally recruited positively connected regions involved in interoception and motor intention perception (insula; Craig and Craig 2009), intentional action production (IFGop; Zapparoli et al. 2018), intention comprehension (IPS and IPL; Blakemore and Decety 2001), and temporal processing related to actions (vermis, especially areas VIII and VIIIa; Rao et al. 2001). Communicative intention comprehension as a function of expertise in spatially shuffled PLD recruited very similar brain areas at the whole-brain level compared with temporal cropping, but with larger clusters. This result could be explained by an advantage in assessing complex visual inputs in violinists compared to non-violinists for assessing instrumental performance (Griffiths and Reay 2018). Specifically, in the spatially altered condition, violinists made greater use of bilateral regions such as IPL and IPS. While IPL helps establish a stable body-centered reference system for movement planning that uses visual and kinesthetic information, IPS is responsible for mental rotation, especially in creative processes such as music composition (Wöllner 2017). Both regions helped violinists make sense of the altered visual information, reconstruct a stable representation of the expressive gestures and associated intention. In addition, general task performance as a group-level covariate constrained our whole-brain results and showed a difference between left and right IPS, with the latter showing a larger cluster of increased activity as a function of task performance in experts vs. controls. This suggests a specific role for the right IPS region in the context of interindividual differences in performance, and indeed this region has been reported to contribute to interpersonal synchronization in the context of actions (Bhat et al. 2017). In addition, the inferior parietal cortex plays a role in discriminating between self-generated actions and actions generated by others. The right inferior parietal cortex is activated when participants mentally simulate actions from the perspective of another person but not from their own perspective (Ruby and Decety 2001). Because this distinction between self and other is a key process for understanding intentions in musical ensemble (Keller et al. 2016), we propose that more accomplished musicians use their ability to distinguish between self and other to better predict intentions. It is also worth mentioning the role of the superior temporal cortex, particularly the posterior STS, which receives important information from the dorsal visual pathway but also from the ventral visual pathway and therefore has a dual role in both perception for identification and action perception (Blakemore and Decety 2001). It was therefore surprising that our data showed no activity in the STS for biological motion perception—comparing piano vs. forte (Blakemore and Decety 2001; Beauchamp 2015), especially since the contrast included correctly assessed PLD. Indeed, posterior STS activity was predicted by performance in a biological movement task (Herrington et al. 2011), and the same region was also shown to communicate with the left cerebellum (Sokolov et al. 2012), several subregions of which showed increased activity in our data, particularly for spatially shuffled PLD that were correctly assessed by violinists. However, STS activity was increased in altered perceptual conditions, and it was interesting to observe greater activity of the right posterior STS for temporally cropped—and not spatially shuffled—PLD in violinists compared with control participants. This result may help to specify the active window of the posterior STS’ function in biological motion perception, namely that this region is activated early in experts and leads to accurate evaluation even when stimulation is present for only a short period of time. However, such assumptions should be tested in more detail using imaging techniques with higher temporal resolution. In addition, functional connectivity analyses revealed coupled temporal cortices (anterior STG, MTG, ITG; posterior MTG, ITG) and negatively coupled connectivity in IFGop, brainstem, and posterior cingulate cortex. The coupled networks showed processing of biological motion independent of motor information (superior and middle temporal cortex; Rizzolatti and Sinigaglia 2010) and attribution of intentions to spatially displaced stimuli (posterior STG; Lee et al. 2012), regions interestingly known for their feedforward connections to the IPS and IPL (Rizzolatti and Sinigaglia 2010). Negatively coupled functional connectivity in turn emphasized intentional action production or unusual action intention processing (IFGop; Zapparoli et al. 2018) and movement initiation and control (brainstem and cerebellum; Rao et al. 2001; Nandi et al. 2002).

While our data shed new light on intention decoding and highlighted additional behavioral contexts that favor experts over non-experts, several limitations should be considered. First, the sample size could have been larger, although it was difficult to recruit several additional violinists who would meet our inclusion criteria. Second, previous studies have highlighted structural brain differences between violinists and non-violinists (Gaser and Schlaug 2003), and therefore, we could have further explored our results by, for example, capturing diffusion tensor imaging to better characterize anatomical variations among our participants. Third, our stimuli contained only point-light representations of violinists, which limits our conclusions regarding other types of expertise (e.g., professional athletes or dancers). However, we chose to study communicative intention in music for its ecological validity and rigorous experimental approach (D’Ausilio et al. 2015). Fourth, although both spatial and temporal changes reflect potential real-world situations, other types of stimulus modification, such as modifying rhythmicity or adding sublevels of visual shuffling, could have been used to further specify the influence of expertise on more subtle perceptual changes and their effect on decoding communicative intent. Fifth, while we used a group-level performance covariate to characterize interindividual differences in ratings of communicative intent between groups, the task-related fMRI data were modeled using only trials with correct intention evaluation. The reason for this decision was that we were primarily interested in correct ratings of communicative intention by experts compared with non-expert participants—but this decision has the major drawback of setting aside variance explained by incorrect ratings, which hinders our interpretation of fMRI results compared with behavioral results. The use of task-specific computational modeling of communicative intent using model-based fMRI analysis could circumvent such problems by the inclusion of every trial and should be a method of choice in the future (Lebreton et al. 2019). Finally, the use of functional—rather than effective—connectivity can be criticized, as Pearson’s correlations between regions do not allow for a test of direct, causal relationship(s) between the regions of interest compared to partial correlations or multivariate regressions (Reid et al. 2019).

Considering our behavioral and neuroimaging data, as well as study limitations, our results suggest a strong role of expertise in understanding and predicting actions and communicative intentions. This claim is especially true under altered perceptual conditions, namely, visually or temporally altered stimuli, and this advantage of violinists over non-violinists would rely on regions of the frontoparietal network, in addition to various areas of the cerebellum, basal ganglia, and brainstem. Such neural systems could also play a role in numerous other conditions in everyday social interactions, as humans are experts at predicting others as they interact with them on a daily basis.

## Data availability statement

The datasets generated during and/or analyzed during the current study are available from the corresponding author on request.

## Code availability statement

The codes used to analyze the data of the current study are available from the corresponding author on request.

## Funding

A project grant from the Swiss National Science Foundation (51NF40–104897—DG). The EU ICT SIEMPRE project gratefully acknowledges financial support from the Future and Emerging Technologies (FET) program within the European Commission’s Seventh Framework Programme for Research (under FET-Open grant number 250026–2).

## Notes

We thank Bruno Bonet of the Brain and Behavior Laboratory of the Medical Center of the University Hospital of Geneva for his assistance in data collection. Finally, we would like to thank the violinists of the Quartetto di Cremona for recording the stimuli and the contribution of violinist experts Chiara Noera and Florence Malgoire for their valuable suggestions. *Conflict of Interest:* None declared.

## Supplementary Material

SupplementaryMaterial_Ceravolo_CCCa_tgab031
