# Supplementary material for: Frontoparietal, Cerebellum Network Codes for Accurate Intention Prediction in Altered Perceptual Conditions
Source: Cereb Cortex Commun. 2021 Apr 23;2(2):tgab031. doi: 10.1093/texcom/tgab031 (PMC8190560; doi:10.1093/texcom/tgab031)
Supplement: SupplementaryMaterial_Ceravolo_CCCa_tgab031 [file supplementarymaterial_ceravolo_ccca_tgab031.pdf]

Supplementary Table 1

Model statistics

Model (participant and sex as random effect)	Information Criterion		Statistics			
	AIC	BIC	Chisq(1, N = 35)	Pr(FDRadj)	R^2_m	R^2_c
Expertise*Nuance	5916.6	5957.4	107.76	<0.001	0.18	0.38
Expertise+Nuance	6022.3	6056.4				
Expertise*Temporal cropping	6265.8	6306.7	84.964	<0.001	0.16	0.32
Expertise+Temporal cropping	6348.7	6382.8				
Expertise*Spatial shuffling	6120.7	6161.6	60.479	<0.001	0.21	0.37
Expertise+Spatial shuffling	6179.2	6213.2				

Each model encompassing the interaction effect between the expertise and one of the three conditions outperformed the corresponding model with only the main effect. The random intercept effect captures the variance induced by every participant and every sex. This indicates the importance of the interaction effect in characterizing the variance of the dataset.

Supplementary Table 2

Model statistics

Model (participant as random effect)	Information Criterion		Statistics			
	AIC	BIC	Chisq(1, N = 35)	Pr(FDRadj)	R^2_m	R^2_c
Expertise*Nuance	5914.6	5948.6	107.76	<0.001	0.18	0.38
Expertise+Nuance	6020.3	6047.6				
Expertise*Temporal cropping	6263.8	6297.8	84.964	<0.001	0.16	0.32
Expertise+Temporal cropping	6346.7	6374				
Expertise*Spatial shuffling	6118.7	6152.7	60.479	<0.001	0.21	0.37
Expertise+Spatial shuffling	6177.2	6204.4				

Each model encompassing the interaction effect between the expertise and one of the three conditions outperformed the corresponding model with only the main effect. The random intercept effect captures the variance induced by every participant. This indicates the importance of the interaction effect in characterizing the variance of the dataset.

Supplementary Table 3

Contrast statistics

Model	Conditions	Statistics	
		Chisq(1, N = 35)	Pr(FDRadj)
Expertise*Nuance	Piano (Mus. > Cont.)	39.578	<0.001
Expertise*Nuance	Forte (Mus. > Cont.)	4.9037	0.027
Expertise*Nuance	Cont. (Piano > Forte)	369.59	<0.001
Expertise*Nuance	Mus. (Piano > Forte)	17.186	<0.001
Expertise*Nuance	Mus. > Cont. & Piano > Forte	107.83	<0.001
Expertise*Temporal cropping	Temp. crop. (Mus. > Cont.)	10.484	0.001
Expertise*Temporal cropping	Temp. unmod. (Mus. > Cont.)	47.993	<0.001
Expertise*Temporal cropping	Cont. (Temp. crop. > unmod.)	7.5669	0.006
Expertise*Temporal cropping	Mus. (Temp. crop. > unmod.)	87.865	<0.001
Expertise*Temporal cropping	Mus. > Cont. & Temp. crop. > unmod.	82.462	<0.001
Expertise*Spatial shuffling	Spat. shuf. (Mus. > Cont.)	14.29	<0.001
Expertise*Spatial shuffling	Spat. unmod. (Mus. > Cont.)	47.935	<0.001
Expertise*Spatial shuffling	Cont. (Spat. shuf. > unmod.)	33.347	<0.001
Expertise*Spatial shuffling	Mus. (Spat. shuf. > unmod.)	179.04	<0.001
Expertise*Spatial shuffling	Mus. > Cont. & Spat. shuf. > unmod.	57.604	<0.001

Contrasts between the different conditions, groups, and their interaction in each computed model. The contrasts and associated figures (Fig 1) show that musicians outperformed control participants in accurately recognizing piano and forte across all conditions. It also highlights the difficulties in recognizing piano nuance, temporally cropped excerpts, and spatially shuffled excerpts.

Supplementary Table 4

Staistics: p-values adjusted for search volume

Model	Region of interest (MNI mm [x,y,z])	Cluster size (k)	peak-level
			T-test
Expertise*Nuance	preSMA (0, 27, 45)	125	t(1,35) = 5.17, p = 0.005
Expertise*Nuance	DLPFC (-42, 12, 30)	15	t(1,35) = 4.11, p = 0.019
Expertise*Spatial shuffling	L INS (-27, 27, -3)	652	t(1,35) = 7.43, p <0.001
Expertise*Spatial shuffling	DLPFC (-45, 27, 24)		t(1,35) = 5.87, p <0.001
Expertise*Spatial shuffling	R INS (33, 27, -3)	943	t(1,35) = 7.31, p <0.001
Expertise*Spatial shuffling	OTC (-36, -72, 15)	1740	t(1,35) = 6.01, p <0.001
Expertise*Spatial shuffling	L IPS (-18, -66, 42)		t(1,35) = 5.71, p <0.001
Expertise*Spatial shuffling	R IPS (33, -39, 51)	984	t(1,35) = 5.89, p <0.001
Expertise*Spatial shuffling	preSMA (9, 21, 42)	437	t(1,35) = 4.72, p <0.001
Expertise*Spatial shuffling	R SMA (15, 0, 66)	295	t(1,35) = 4.5, p <0.001
Expertise*Spatial shuffling	Vermis 4 5 (9, -54, -24)	263	t(1,35) = 4.22, p <0.001
Expertise*Temporal cropping	IPL (-36, -33, 51)	433	t(1,35) = 5.19, p = 0.003
Expertise*Temporal cropping	L pMTG (-48, -72, 9)	138	t(1,35) = 5.17, p = 0.003
Expertise*Temporal cropping	R pMTG (51, -57, 12)	190	t(1,35) = 4.79, p = 0.005
Expertise*Temporal cropping	L DLPFC (-42, 30, 30)	197	t(1,35) = 4.6, p = 0.005
Expertise*Temporal cropping	L Post Central Gyrus (-57, -18, 36)	74	t(1,35) = 4.5, p = 0.006
Expertise*Temporal cropping	L Cereb 8 (-15, -57, -36)	23	t(1,35) = 4.14, p = 0.01
Expertise*Temporal cropping	R IFG (54, 33, 18)	91	t(1,35) = 4.1, p = 0.01
Expertise*Temporal cropping	R INS (39, 24, -3)	32	t(1,35) = 4.09, p = 0.01
Expertise*Temporal cropping	L SMA (-9, -3, 66)	15	t(1,35) = 3.75, p = 0.016
Expertise*Temporal cropping	R preSMA (6, 24, 51)	27	t(1,35) = 3.73, p = 0.016
Covariate Expertise*Spatial shuffling	R INS (39, 21, -6)	741	t(1,35) = 6.14, p <0.001
Covariate Expertise*Spatial shuffling	R DLPFC (39, 15, 24)		t(1,35) = 4.94, p <0.001
Covariate Expertise*Spatial shuffling	L INS (-27, 27, -6)	332	t(1,35) = 5.97, p <0.001
Covariate Expertise*Spatial shuffling	L DLPFC (-48, 27, 27)		t(1,35) = 5.23, p <0.001
Covariate Expertise*Spatial shuffling	OTC (-45, -63, -3)	154	t(1,35) = 5.03, p <0.001
Covariate Expertise*Spatial shuffling	R IPS (33, -39, 51)	160	t(1,35) = 4.78, p = 0.001
Covariate Expertise*Spatial shuffling	L IPS (-27, -69, 30)	183	t(1,35) = 4.72, p = 0.001
Covariate Expertise*Spatial shuffling	preSMA (0, 30, 45)	161	t(1,35) = 4.56, p = 0.001
Covariate Expertise*Spatial shuffling	L Sup Marg (-63, -30, 27)	38	t(1,35) = 4.05, p = 0.001
Covariate Expertise*Spatial shuffling	R Caudate (9, 12, 6)	33	t(1,35) = 3.99 p = 0.001
Covariate Expertise*Spatial shuffling	R Temp Mid (39, -60, 9)	24	t(1,35) = 3.91 p = 0.001
Covariate Expertise*Spatial shuffling	L Cereb 7b (-9, -72, -42)	24	t(1,35) = 3.73 p = 0.001
Covariate Expertise*Temporal cropping	IPL (-36, -36, 48)	1121	t(1,35) = 5.56, p = 0.001
Covariate Expertise*Temporal cropping	L pMTG (-48, -75, 9)	220	t(1,35) = 4.66, p = 0.003
Covariate Expertise*Temporal cropping	L INS (-30, 27, 0)	113	t(1,35) = 4.52, p = 0.004
Covariate Expertise*Temporal cropping	L RO (-45, 6, 15)	299	t(1,35) = 4.47, p = 0.004
Covariate Expertise*Temporal cropping	L SMA (-9, -21, 48)	173	t(1,35) = 4.31, p = 0.004
Covariate Expertise*Temporal cropping	R IFG pars tri (51, 36, 21)	39	t(1,35) = 3.97, p = 0.007
Covariate Expertise*Temporal cropping	R Cereb 6 (21, -48, -27)	65	t(1,35) = 3.96, p = 0.007
Covariate Expertise*Temporal cropping	L Cereb 8 (-15, -57, -33)	29	t(1,35) = 3.96, p = 0.007
Covariate Expertise*Temporal cropping	R INS (33, 30, -6)	42	t(1,35) = 3.86, p = 0.009
Covariate Expertise*Temporal cropping	L Sup Marg (-48, -39, 27)	32	t(1,35) = 3.7, p = 0.011
Covariate Expertise*Temporal cropping	R pMTG (42, -63, 3)	148	t(1,35) = 3.55, p = 0.015

Brain regions showing significant greater activity for the three contrasts: musician > control * piano > forte, musician > control * temporally cropped > unmodified, musician > control * spatially shuffled > unmodified. For all contrasts, significance is corrected at the peak using p<.05 false discovery rate (FDR). Minimal voxel size k = 10.
aITG: inferior temporal gyrus, anterior part; aMTG: medial temporal gyrus, anterior part; aSTG: supperior temporagl gyrus, anterior part; aTFus: temporal fusiform, anterior part; Cereb6: cerebellum 6; Cereb1: cerebellum 1; Cereb3: cerebellum 3; Cereb8: cerebellum 8; DLFC: dorso lateral frontal cortex; DLPFC: dorso lateral prefrontal cortex; FO: frontal operculum; FP: frontal pole; IFG oper: inferior frontal gyrus operculum; IFG pars tri: inferior frontal gyrus pars triangularis; INS: insula; IPL: inferior parietal lobule; IPS: inferior parietal sulcus; L: left; LG: lingual gyrus; MFC: medial frontal cortex; MTG: medial temporal gyrus; OTC: occipito-temporal cortex; PFC: prefrontal cortex; pITG: inferior temporal gyrus, posterior part; pMTG: medial temporal gyrus, posterior part; Post Central Gyrus: posterior central gyrus; Post Cing: posterior cingulate; PostCG: posterior cingulate gyrus; preSMA: pre supplementary motor area; pSMG: superior medial gyrus, posterior part; pTFusC: temporal fusiform cortex, posterior part; R: right; RO: rolandic operculum; SMA: superior motor area; SMG: superior medial gyrus; SPL: superior parietal lobule; SubCalC: subcallosal cortex; SupMarg: supramarginal; toITG: inferior temporal gyrus, temporooccipital part; toMTG: medial temporal gyrus, temporooccipital part; Temp Mid: temporal middle; Ver7: Vernis 7; Ver8: Vernis 8;

Supplementary Table 5

ANOVA Betas for each region of interest

Model	Region of interest (MNI mm [x,y,z])	F-test
Expertise*Nuance	DLPFC (-42 12 30)	F(1,35) = 3.16, p = 0.08
Expertise*Nuance	preSMA (0 27 45)	F(1,35) = 9.94, p = 0.002
Expertise*Spatial shuffling	DLPFC (-45 27 24)	F(1,35) = 10.92, p = 0.002
Expertise*Spatial shuffling	L INS (-27 27 -3)	F(1,35) = 8.1, p = 0.006
Expertise*Spatial shuffling	L IPS (-18 -66 42)	F(1,35) = 5.32, p = 0.024
Expertise*Spatial shuffling	OTC (-36 -72 15)	F(1,35) = 2.14, p = 0.148
Expertise*Spatial shuffling	preSMA (9 21 42)	F(1,35) = 3.9, p = 0.052
Expertise*Spatial shuffling	R INS (33 27 -3)	F(1,35) = 13.78, p < 0.001
Expertise*Spatial shuffling	R IPS (33 -39 51)	F(1,35) = 3.66, p = 0.06
Expertise*Temporal cropping	IPL (-36 -33 51)	F(1,35) = 2.41, p = 0.125
Expertise*Temporal cropping	L pMTG (-48 -72 9)	F(1,35) = 0.6, p = 0.441
Expertise*Temporal cropping	R pMTG (51 -57 12)	F(1,35) = 1.11, p = 0.295
Expertise*Temporal cropping	R preSMA (6 24 51)	F(1,35) = 2.2, p = 0.143
Expertise*Temporal cropping	L DLPFC (-42 30 30)	F(1,35) = 5.22, p = 0.026
Expertise*Temporal cropping	L Post Central Gyrus (-57 -18 36)	F(1,35) = 1.84, p = 0.18
Expertise*Temporal cropping	L Cereb 8 (-15 -57 -36)	F(1,35) = 2.64, p = 0.109
Expertise*Temporal cropping	R IFG pars tri (54 33 18)	F(1,35) = 2.37, p = 0.128
Expertise*Temporal cropping	R INS (39 24 -3)	F(1,35) = 2.05, p = 0.157
Expertise*Temporal cropping	L SMA (-9 -3 66)	F(1,35) = 1.84, p = 0.179

ANOVA comparing the interaction between the expertise and the different conditions for the beta extracted from the SPM.mat of first-level analysis.

aITG: inferior temporal gyrus, anterior part; aMTG: medial temporal gyrus, anterior part; aSTG: supperior temporagl gyrus, anterior part; aTFus: temporal fusiform, anterior part; Cereb6: cerebellum 6; Cereb1: cerebellum 1; Cereb3: cerebellum 3; Cereb8: cerebellum 8; DLFC: dorso lateral frontal cortex; DLPFC: dorso lateral prefrontal cortex; FO: frontal operculum; FP: frontal pole; IFG oper: inferior frontal gyrus operculum; IFG pars tri: inferior frontal gyrus pars triangularis; INS: insula; IPL: inferior parietal lobule; IPS: inferior parietal sulcus; L: left; LG: lingual gyrus; MFC: medial frontal cortex; MTG: medial temporal gyrus; OTC: occipito-temporal cortex; PFC: prefrontal cortex; pITG: inferior temporal gyrus, posterior part; pMTG: medial temporal gyrus, posterior part; Post Central Gyrus: posterior central gyrus; Post Cing: posterior cingulate; PostCG: posterior cingulate gyrus; preSMA: pre supplementary motor area; pSMG: superior medial gyrus, posterior part; pTFusC: temporal fusiform cortex, posterior part; R: right; RO: rolandic operculum; SMA: superior motor area; SMG: superior medial gyrus; SPL: superior parietal lobule; SubCalC: subcallosal cortex; SupMarg: supramarginal; toITG: inferior temporal gyrus, temporooccipital part; toMTG: medial temporal gyrus, temporooccipital part; Ver7: Vernis 7; Ver8: Vernis 8;

Supplementary Table 6

Connectivity Statistics

Model	Seed	Target	Statistics
Expertise*Nuance	aTFusC L	aMTG L	T(33) = 4.06, p = 0.043
Expertise*Nuance	Cereb3 L	Cereb10 L	T(33) = 4.60, p = 0.009
Expertise*Nuance	SubCalC	MFC	T(33) = -4.13, p = 0.027
Expertise*Nuance	SubCalC	PostCing	T(33) = -3.98, p = 0.027
Expertise*Nuance	DLPFC L	IPS L	T(33) = -4.19, p = 0.03
Expertise*Nuance	DLPFC L	toITGl	T(33) = -3.89, p = 0.034
Expertise*Nuance	Brain-Stem	LG R	T(33) = 4.42, p = 0.015
Expertise*Nuance	Brain-Stem	LG L	T(33) = 3.82, p = 0.032
Expertise*Nuance	Brain-Stem	Cereb8 L	T(33) = 3.78, p = 0.032
Expertise*Nuance	MFC	SubCalC	T(33) = -4.13, p = 0.035
Expertise*Nuance	Putamen L	aMTG R	T(33) = 4.19, p = 0.029
Expertise*Nuance	FP L	FO R	T(33) = -4.01, p = 0.05
Expertise*Nuance	aMTG R	Putamen L	T(33) = 4.19, p = 0.029
Expertise*Nuance	LG R	Brain-Stem	T(33) = 4.42, p = 0.015
Expertise*Nuance	aMTG L	aTFusC L	T(33) = 4.06, p = 0.043
Expertise*Nuance	aMTG L	pTFusC R	T(33) = 3.77, p = 0.048
Expertise*Nuance	IPS L	DLPFC L	T(33) = -4.19, p = 0.03
Expertise*Nuance	Cereb10 L	Cereb3 L	T(33) = 4.60, p = 0.009
Expertise*Nuance	FO R	FP L	T(33) = -4.01, p = 0.05
Expertise*Spatial shuffling	IFG oper L	PostCG R	T(33) = -4.50, p = 0.012
Expertise*Spatial shuffling	aITG L	pMTG L	T(33) = 3.81, p = 0.038
Expertise*Spatial shuffling	aITG L	aMTG L	T(33) = 3.75, p = 0.038
Expertise*Spatial shuffling	aITG L	SMG R	T(33) = 3.71, p = 0.038
Expertise*Spatial shuffling	aITG L	aSTG L	T(33) = 3.53, p = 0.048
Expertise*Spatial shuffling	pITG R	Brain-Stem	T(33) = -4.30, p = 0.021
Expertise*Spatial shuffling	Brain-Stem	pITG R	T(33) = -4.30, p = 0.021
Expertise*Spatial shuffling	PostCG R	IFG oper L	T(33) = -4.50, p = 0.012
Expertise*Temporal cropping	Ver8	IFG oper L	T(33) = 5.31, p = 0.001
Expertise*Temporal cropping	Ver8	DLPFC	T(33) = 4.58, p = 0.004
Expertise*Temporal cropping	Ver8	toMTGl	T(33) = 4.52, p = 0.004
Expertise*Temporal cropping	Ver8	pSMGl	T(33) = 4.02, p = 0.012
Expertise*Temporal cropping	Ver8	IPS R	T(33) = 3.51, p = 0.037
Expertise*Temporal cropping	Ver8	IFG oper R	T(33) = 3.47, p = 0.037
Expertise*Temporal cropping	Ver8	INS L	T(33) = 3.35, p = 0.045
Expertise*Temporal cropping	Ver8	DLPFC L	T(33) = 3.29, p = 0.045
Expertise*Temporal cropping	SPLl	Ver7	T(33) = 4.04, p = 0.046
Expertise*Temporal cropping	IFG oper L	Ver8	T(33) = 5.31, p = 0.001
Expertise*Temporal cropping	to MTG L	Ver8	T(33) = 4.52, p = 0.012
Expertise*Temporal cropping	Ver7	SPL L	T(33) = 4.04, p = 0.046
Expertise*Temporal cropping	DLPFC	Ver8	T(33) = 4.58, p = 0.01
Expertise*Temporal cropping	pSMGl	Ver8	T(33) = 4.02, p = 0.048

Statistics associated with the different connections in the connectivity analysis.
P value is FDR corrected at p<.05; aITG: inferior temporal gyrus, anterior part; aMTG: medial temporal gyrus, anterior part; aSTG: supperior temporagl gyrus, anterior part; aTFus: temporal fusiform, anterior part; Cereb6: cerebellum 6; Cereb1: cerebellum 1; Cereb3: cerebellum 3; Cereb8: cerebellum 8; DLFC: dorso lateral frontal cortex; DLPFC: dorso lateral prefrontal cortex; FO: frontal operculum; FP: frontal pole; IFG oper: inferior frontal gyrus operculum; IFG pars tri: inferior frontal gyrus pars triangularis; INS: insula; IPL: inferior parietal lobule; IPS: inferior parietal sulcus; L: left; LG: lingual gyrus; MFC: medial frontal cortex; MTG: medial temporal gyrus; OTC: occipito-temporal cortex; PFC: prefrontal cortex; pITG: inferior temporal gyrus, posterior part; pMTG: medial temporal gyrus, posterior part; Post Cing: posterior cingulate; PostCG: posterior central gyrus; preSMA: pre supplementary motor area; pSMG: superior medial gyrus, posterior part; pTFusC: temporal fusiform cortex, posterior part; R: right; RO: rolandic operculum; SMA: superior motor area; SMG: superior medial gyrus; SPL: superior parietal lobule; SubCalC: subcallosal cortex; SupMarg: supramarginal; toITG: inferior temporal gyrus, temporooccipital part; toMTG: medial temporal gyrus, temporooccipital part; Ver7: Vernis 7; Ver8: Vernis 8;

Supplementary Figure 1

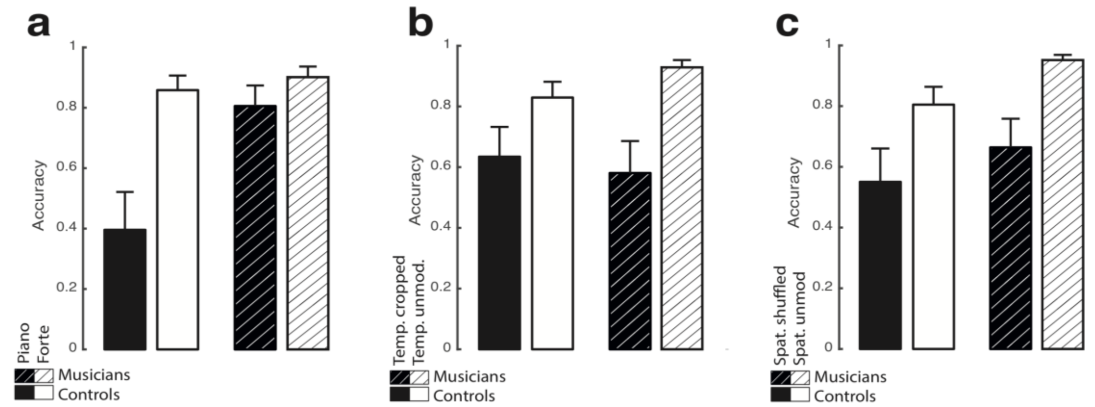

Behavioral performance of both groups for every interaction when the sex is captured by random intercept effect. (**a**) Averaged performance per group for piano vs. forte piece dynamics. (**b**) Averaged performance per group for piano vs. forte piece dynamics in temporally cropped vs. temporally unmodified sequences. (**c**) Averaged performance per group for piano vs. forte piece dynamics in spatially shuffled vs. spatially unmodified sequences. The present results highlight the behavioral performance of the participants when the variance associated with sex is captured by the model. Similar patterns can be observed with or without sex being captured by random effects.

Supplementary Figure 2

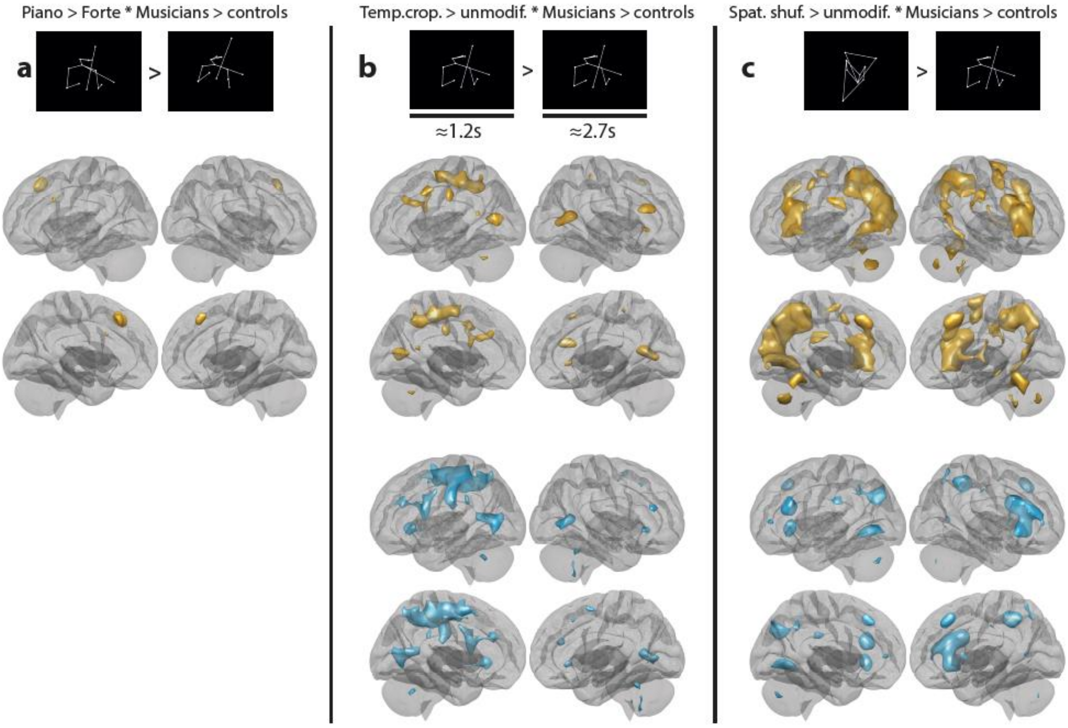

Activations represented in volumes in the brain for the different conditions. (a) Piano>Forte * Musicians > Controls. (b) Temporally cropped>Temporally unmodified * Musicians > Controls. (c) Spatially shuffled>Spatially unmodified * Musicians > Controls. Gold clusters are the effect of condition contrasts while teal clusters reflect the interaction with the group-level general task performance covariate. $p<.05$, FDR corrected at the peak level.

Supplementary Figure 3

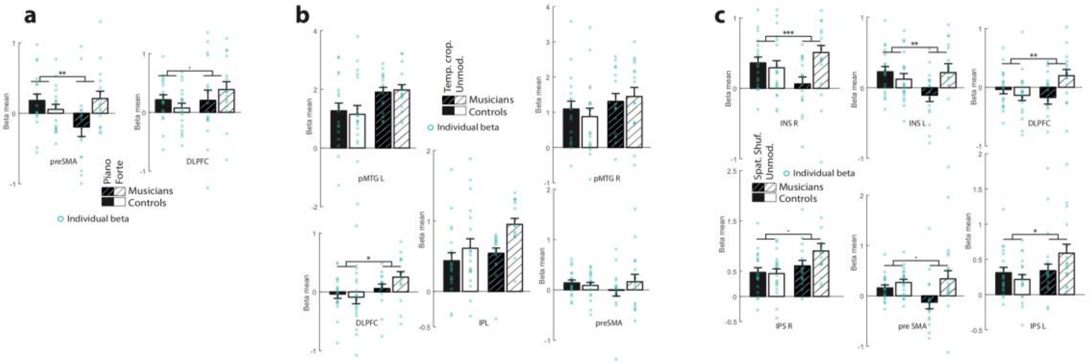

(**a**) Beta values of the general linear model (GLM) for piano and forte sequences for musicians and control participants. (**b**) Beta values of the GLM for temporally cropped and unmodified sequences for musicians and control participants. (**c**) Beta values of the GLM for spatially shuffled and unmodified sequences for musicians and control participants.

aITG: inferior temporal gyrus, anterior part; aMTG: medial temporal gyrus, anterior part; aSTG: supperior temporagl gyrus, anterior part; aTFus: temporal fusiform, anterior part; Cereb6: cerebellum 6; Cereb1: cerebellum 1; Cereb3: cerebellum 3; Cereb8: cerebellum 8; DLFC: dorso lateral frontal cortex; DLPFC: dorso lateral prefrontal cortex; FO: frontal operculum; FP: frontal pole; IFG oper: inferior frontal gyrus operculum; IFG pars tri: inferior frontal gyrus pars triangularis; INS: insula; IPL: inferior parietal lobule; IPS: inferior parietal sulcus; L: left; LG: lingual gyrus; MFC: medial frontal cortex; MTG: medial temporal gyrus; OTC: occipito-temporal cortex; PFC: prefrontal cortex; pITG: inferior temporal gyrus, posterior part; pMTG: medial temporal gyrus, posterior part; Post Central Gyrus: posterior central gyrus; Post Cing: posterior cingulate; PostCG: posterior cingulate gyrus; preSMA: pre supplementary motor area; pSMG: superior medial gyrus, posterior part; pTFusC: temporal fusiform cortex, posterior part; R: right; RO: rolandic operculum; SMA: superior motor area; SMG: superior medial gyrus; SPL: superior parietal lobule; SubCalC: subcallosal cortex; SupMarg: supramarginal; toITG: inferior temporal gyrus, temporooccipital part; toMTG: medial temporal gyrus, temporooccipital part; Ver7: Vernis 7; Ver8: Vernis 8;

Supplementary Figure 4

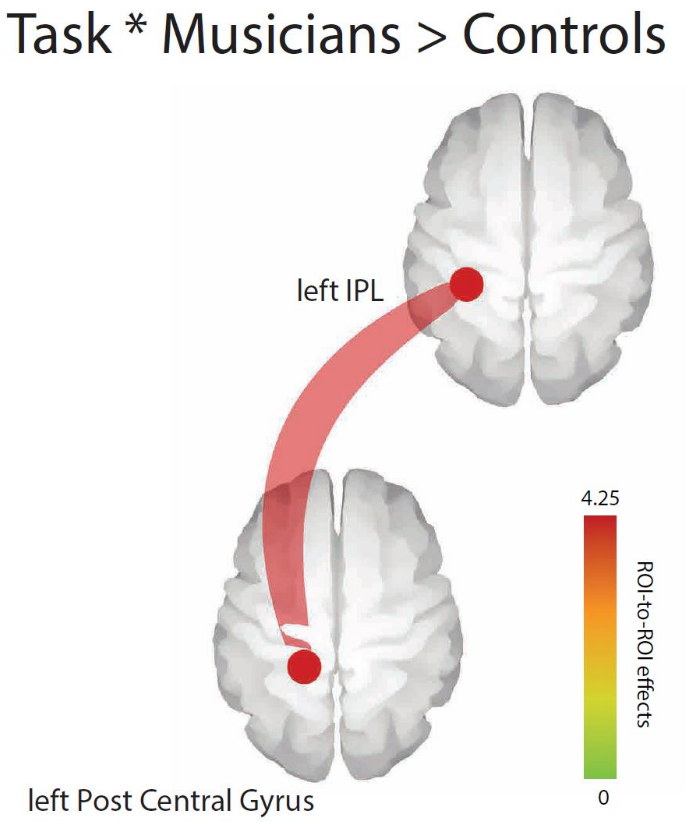

Functional connectivity between regions of interest highlighted in the GLM analysis for the main effect of the task.
